# Mendelian randomization analyses suggest a causal role for circulating GIP and IL-1RA levels in homeostatic model assessment-derived measures of β-cell function and insulin sensitivity in Africans without type 2 diabetes

**DOI:** 10.1186/s13073-023-01263-7

**Published:** 2023-12-04

**Authors:** Karlijn A. C. Meeks, Amy R. Bentley, Themistocles L. Assimes, Nora Franceschini, Adebowale A. Adeyemo, Charles N. Rotimi, Ayo P. Doumatey

**Affiliations:** 1 Center for Research on Genomics and Global Health, National Human Genome Research Institute, National Institutes of Health, 12 South Drive Bldg 12A ste 1025, Bethesda, MD 20892-5611 USA; 2 Department of Medicine, Stanford University School of Medicine, Stanford, CA USA; 3 VA Palo Alto Healthcare System, Palo Alto, CA USA; 4 Department of Epidemiology, University of North Carolina, Chapel Hill, NC USA

**Keywords:** Cytokines, Hormones, Type 2 diabetes, Sub-Saharan Africans, Mendelian randomization, Causal inference

## Abstract

**Background:**

In vitro and in vivo studies have shown that certain cytokines and hormones may play a role in the development and progression of type 2 diabetes (T2D). However, studies on their role in T2D in humans are scarce. We evaluated associations between 11 circulating cytokines and hormones with T2D among a population of sub-Saharan Africans and tested for causal relationships using Mendelian randomization (MR) analyses.

**Methods:**

We used logistic regression analysis adjusted for age, sex, body mass index, and recruitment country to regress levels of 11 cytokines and hormones (adipsin, leptin, visfatin, PAI-1, GIP, GLP-1, ghrelin, resistin, IL-6, IL-10, IL-1RA) on T2D among Ghanaians, Nigerians, and Kenyans from the Africa America Diabetes Mellitus study including 2276 individuals with T2D and 2790 non-T2D individuals. Similar linear regression models were fitted with homeostatic modelling assessments of insulin sensitivity (HOMA-S) and β-cell function (HOMA-B) as dependent variables among non-T2D individuals (*n* = 2790). We used 35 genetic variants previously associated with at least one of these 11 cytokines and hormones among non-T2D individuals as instrumental variables in univariable and multivariable MR analyses. Statistical significance was set at 0.0045 (0.05/11 cytokines and hormones).

**Results:**

Circulating GIP and IL-1RA levels were associated with T2D. Nine of the 11 cytokines and hormones (exceptions GLP-1 and IL-6) were associated with HOMA-S, HOMA-B, or both among non-T2D individuals. Two-stage least squares MR analysis provided evidence for a causal effect of GIP and IL-RA on HOMA-S and HOMA-B in multivariable analyses (GIP ~ HOMA-S *β* =  − 0.67, *P*-value = 1.88 × 10^−6^ and HOMA-B *β* = 0.59, *P*-value = 1.88 × 10^−5^; IL-1RA ~ HOMA-S *β* =  − 0.51, *P*-value = 8.49 × 10^−5^ and HOMA-B *β* = 0.48, *P*-value = 5.71 × 10^−4^). IL-RA was partly mediated via BMI (30-34%), but GIP was not. Inverse variance weighted MR analysis provided evidence for a causal effect of adipsin on T2D (multivariable OR = 1.83, *P*-value = 9.79 × 10^−6^), though these associations were not consistent in all sensitivity analyses.

**Conclusions:**

The findings of this comprehensive MR analysis indicate that circulating GIP and IL-1RA levels are causal for reduced insulin sensitivity and increased β-cell function. GIP’s effect being independent of BMI suggests that circulating levels of GIP could be a promising early biomarker for T2D risk. Our MR analyses do not provide conclusive evidence for a causal role of other circulating cytokines in T2D among sub-Saharan Africans.

**Supplementary Information:**

The online version contains supplementary material available at 10.1186/s13073-023-01263-7.

## Background

The global burden of type 2 diabetes (T2D) is high and rising and disproportionally affects African-ancestry populations. In the USA, African Americans have a 1.6 times higher prevalence of T2D than European Americans [1] and sub-Saharan African migrants in Europe are nearly three times more likely to have T2D compared with Europeans [2]. Furthermore, a steady rise in T2D prevalence is observed in sub-Saharan Africa [3].

The reasons for the disproportionate burden of T2D among African-ancestry populations are not completely understood. Inflammatory processes have been implicated in the development and progression of T2D [4]. Such an inflammatory state is characterized by increased circulating levels of pro-inflammatory cytokines and hormones and reduced levels of anti-inflammatory ones [5]. Cytokines and hormones relevant to T2D can be classified into different clusters based on their site of production, including those produced by adipose tissue (such as adipsin, leptin, visfatin, and plasminogen activator inhibitor-1(PAI-1)), those produced by the gut (such as glucose-dependent insulinotropic peptide (GIP), glucagon-like peptide-1 (GLP-1), and ghrelin), and those produced by immune cells (such as resistin, interleukin 6 (IL-6), interleukin 10 (IL-10), and interleukin 1 receptor antagonist (IL-1RA)). Most studies on these circulating cytokines and hormones have been performed in vitro and in vivo [6, 7]. The observational studies available have associated a limited number of cytokines and hormones with insulin sensitivity and T2D in diverse populations [8–11], including African Americans and West Africans [12–15]. However, these studies have focused on a handful of cytokines and hormones, such as adiponectin [8, 13], adipsin [9], PAI-1 [10], IL-6 [11, 12, 14, 15], IL-10 [12, 14], and IL-1RA [12, 14], while analyses on other potentially diabetes-related cytokines and hormones such as visfatin, GIP, GLP-1, and ghrelin are scarce. In addition, cross-sectional observational studies are unable to determine causality, i.e. whether circulating cytokine and hormone levels are a cause or consequence of T2D.

Mendelian randomization (MR) analysis leverages genetic variants as instrumental variables to improve causal inference in observational studies. Specifically, MR studies are less affected by limitations such as unmeasured confounding and reverse causation because of the random distribution of genotypes at conception. MR studies assessing causality for the relationship between cytokines and hormones with T2D are limited. Wang et al. found evidence for a causal effect of leptin levels on T2D in European-ancestry individuals [16], while for IL1-RA, no evidence for a causal effect on glycaemic traits was found [17]. MR studies assessing the effect of other cytokines on T2D are lacking and MR studies for any cytokine or hormone on T2D are absent in sub-Saharan African populations. As levels of diabetes-related cytokines and hormones have been found to differ in African-ancestry individuals compared with European-ancestry individuals [18–21], the causal effect of these cytokines and hormones on T2D risk could also differ. A better understanding of the role of cytokines and hormones in T2D among African-ancestry populations may have important preventive and therapeutic implications.

Here, we aimed to (1) evaluate the association between 11 circulating cytokines and hormones with T2D-related phenotypes, and (2) infer causality in these associations using MR analyses in a population of sub-Saharan Africans.

## Methods

### Study design and data sources

The Africa America Diabetes Mellitus (AADM) study is the longest-running genetic epidemiology study of T2D in sub-Saharan Africa. The study enrolled over 6000 sub-Saharan African adults aged 25 years and above with T2D and adults without T2D (non-T2D individuals) from university medical centres in Nigeria, Ghana, and Kenya. The study design and procedures have been described in detail elsewhere [22–24]. In brief, individuals with T2D were enrolled through major medical centres in three cities in Nigeria (Ibadan, Enugu, and Lagos), two cities in Ghana (Accra, and Kumasi), and in the city of Eldoret in Kenya. Non-T2D individuals were enrolled from surrounding communities of the various participating medical centres. If individuals in the community expressed interest in participating in the study, they were then invited to the study clinic where the formal process of obtaining informed consent took place. Yoruba and Igbo (Nigeria), Akan and Gaa-Adangbe (Ghana), and Luhya, Kikuyu, and Kalenjin (Kenya) were the most common ethnolinguistic groups among the study participants.

### Measurements

Demographic data including age and sex were obtained through structured questionnaires. Height and weight were measured in light clothing and body mass index (BMI) was calculated as weight/height^2^ (kg/m^2^).

Fasting serum and plasma samples were obtained by trained personnel. Fasting glucose concentration was measured in mg/dl using the enzymatic reference method with hexokinase on a Roche analyzer. Eleven circulating cytokines and hormones, namely adipsin, leptin, visfatin, PAI-1, GIP, GLP-1, ghrelin, resistin, IL-6, IL-10, and IL-1RA were measured using multiplex bead-based flow cytometric immunoassays according to the manufacturer’s instructions (Bio-Plex Pro human diabetes: 10-plex, Cat#171A7001M and 2-plex, Cat#171A7002M, Bio-Rad, Inc., Hercules, CA, USA). These commercial kits measure the levels of cytokines and hormones reported to be involved in obesity and diabetes pathophysiology. Data were collected using Bio-Plex 200®System (Luminex Corporation, Austin, TX) equipped with Bio-Plex Manager™ Software (Bio-Rad, Inc., Hercules, CA, USA). IL-6, IL-10, and IL-1RA were analysed in a subset of participants using enzyme-linked immunosorbent assay (ELISA) (Quantikine ELISA, R&D Systems, Minneapolis, MN, USA).

T2D was defined using the American Diabetes Association (ADA) criteria, i.e. a fasting glucose of ≥ 126 mg/dl (≥ 7.0 mmol/L), or an oral glucose tolerance test (OGTT) 2-h post load value of ≥ 200 mg/dl (11.1 mmol/L) on more than one occasion, or the reported use of glucose-lowering medication as prescribed by a physician confirmed by review of clinical records. The updated Homeostatic Model Assessment (HOMA) was used through the University of Oxford HOMA2 calculator to estimate insulin sensitivity (HOMA-S) and β-cell function (HOMA-B). The updated HOMA model is a computer model that derives HOMA-S and HOMA-B as percentages of a normal reference population rather than linear approximations (available from: https://www.dtu.ox.ac.uk/homacalculator/). HOMA-S is the reciprocal of HOMA-IR (insulin resistance) as calculated using the linear set of equations. Both HOMA-S and HOMA-B were calculated for non-T2D individuals only as the measures are deemed not to be valid in the presence of T2D.

### Association analyses with T2D and HOMA measures

**Fig. 1:**
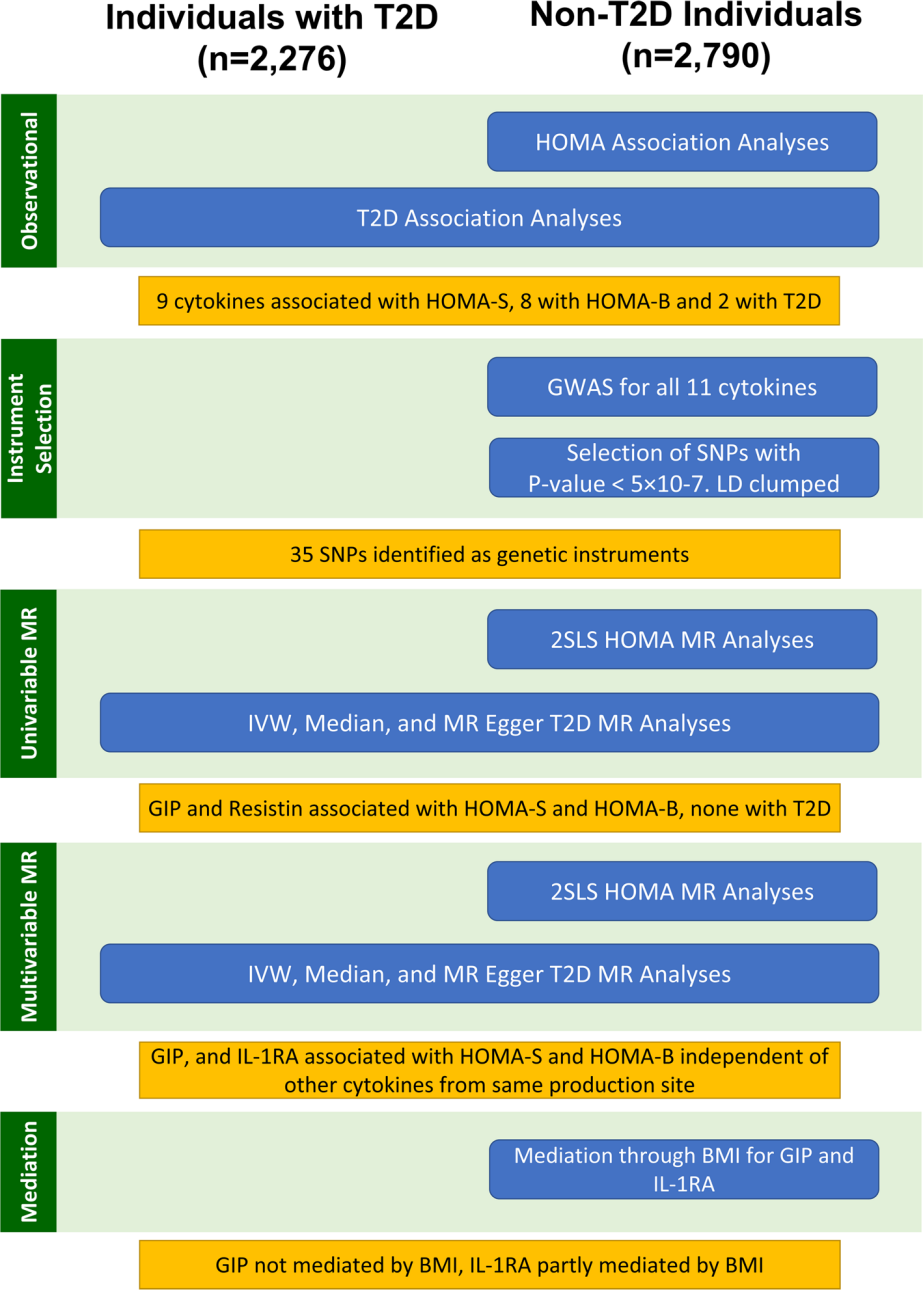
Overview of analyses performed to identify circulating cytokines and hormones causally associated with HOMA-S, HOMA-B, and/or T2D. T2D = type 2 diabetes, HOMA = homeostatic modelling assessment of insulin sensitivity and insulin secretion, GWAS = genome-wide association study, LD = linkage disequilibrium, 2SLS = two-stage least squares, IVW = inverse variance weighted, MR = Mendelian randomization, BMI = body mass index

A total of 5066 participants were available for association analysis who had at least one of the 11 cytokines and hormones measured as well as data on T2D status (Fig. 1). We used G*power to calculate a post hoc power of 92.6% for an OR of 1.1 and 99.9% to detect an OR of 1.2 at an alpha of 0.05 [25]. A total of 2790 non-T2D individuals were available for the analyses with HOMA-S and HOMA-B. We performed complete-case analyses for all three outcomes, i.e. individuals with missing values on any variable in the model were excluded from the analyses for that specific outcome. An overview of all analyses performed can be found in Fig. 1.

Analyses were performed using the R statistical computing platform (version 4.2.2) and R studio (version 2022.12.0) [26, 27]. The *bestNormalize* R package was used to find the best-performing transformation for all cytokines, hormones, and both HOMA measures [28]. The ordered quantile (ORQ) transformation was found to be optimal and was applied to the cytokine/hormone and HOMA measures. We subsequently fitted linear regression models adjusted for age, sex, BMI, and recruitment country among non-T2D individuals to regress each of the cytokines and hormones on HOMA-S and HOMA-B. Logistic regression models were fitted to assess the association between each of the cytokines and hormones with T2D status with adjustment for age, sex, BMI, and recruitment country.

### Instrument selection

For each cytokine and hormone, instruments were derived from previously published genome-wide association analyses among AADM study participants without T2D [29]. In brief, participants’ samples were genotyped using the Affymetrix Axiom PANAFR SNP array or Illumina’s Multi-Ethnic Global Array (MEGA) [24]. Quality control resulted in a sample-level genotype call rate of at least 0.95 for all samples. The SNP datasets were filtered for missingness per marker (> 0.05), minor allele frequency (< 0.01), and Hardy–Weinberg equilibrium (*P*-value ≤ 1 × 10^ −6^) and imputed using the African Genome Resources Haplotype Reference Panel via the Sanger Imputation Service. Genome-wide quantitative linear regression analyses were performed for all cytokines and hormones separately with adjustment for age, sex, T2D, the first three principal components (PCs), and a genetic relatedness matrix [29]. For instrument selection, we used these summary statistics from which we additionally filtered out SNPs with low imputation quality by excluding SNPs with INFO scores of < 0.8. All SNPs with a genome-wide *P*-value of < 5 × 10^−7^ were subsequently selected as potential instruments. We then grouped these SNPs into genetic loci using pairwise linkage disequilibrium (LD) analysis. This procedure, commonly known as “LD clumping” was performed separately for each cytokine and hormone. The PLINK 1.9 software [30] was used for this purpose, setting an r^2^ threshold of < 0.1 and all unrelated AADM participants as the LD reference. The SNPs with the lowest *P*-value in each clump were selected as independent instruments. We used the *mRnd* online web tool to calculate the power of the MR analyses with these selected instruments for an *α* of 0.05 and a true causal effect size of *β* = 0.2 for the HOMA measures and OR = 1.5 for T2D [31].

### Mendelian randomization analysis

#### Univariable Mendelian randomization analysis

To estimate the univariable effect of each of the cytokines and hormones on the continuous outcomes HOMA-S and HOMA-B, the two-stage least squares (2SLS) method was used through the ivreg function of the *AER* package [32]. In these analyses performed among non-T2D only (*n* = 2790), each cytokine was regressed on its genetic instruments. The fitted values of these regressions were subsequently used in regression with HOMA-S and HOMA-B as dependent variables.

For the univariable T2D MR analysis (*n* = 5066), the inverse variance weighted (IVW) method as implemented in the *MendelianRandomization* R package was used for multi-SNP instruments, while for single-SNP instruments (i.e. IL-6) the Wald ratio was used [33]. We calculated the regression coefficients for the instrument-cytokine/hormone associations in non-T2D individuals using linear regression and the odds ratios (ORs) with corresponding 95% confidence intervals (95% CI) for the instrument-T2D association using logistic regression analysis. This approach was chosen to reduce bias as it has been shown that when instruments are selected in the controls only for a binary outcome, bias follows the same pattern as in a two-sample setting [34]. The IVW method assumes that all genetic variants are valid instruments. We performed several sensitivity analyses using methods that make alternative assumptions to minimize risk of violating a core MR assumption. We used a random effects model for the IVW analyses in addition to the default fixed effects model. The random effects model allows for heterogeneity between the causal estimates by allowing over-dispersion in the model. We also report Cochran’s *Q* and the *I*^2^ statistic for the IVW analyses as an assessment of heterogeneity. In addition, we performed MR-Egger and weighted median analyses. These analyses could only be performed for cytokines and hormones with three or more genetic instruments. MR-Egger corrects for pleiotropy by assuming that the pleiotropic effects of each instrument follow a normal distribution with a mean of zero and a variance that can be estimated from the data [35]. MR-Egger allows for all genetic instruments to have pleiotropic effects but has much lower power than other methods. We report the MR-Egger intercept as a measure for the presence of horizontal pleiotropy. The weighted median method can handle up to 50% of invalid instruments or pleiotropic instruments, as long as the majority of the instruments are valid [36]. We considered concordance across MR methods as robust evidence for causality.

#### Multivariable Mendelian randomization analysis

Several of the eleven circulating cytokines and hormones are biologically related and have shared genetic predictors. To address this situation, we performed multivariable MR analysis in addition to the univariable MR analysis. For the multivariable MR analysis, we considered three clusters of cytokines and hormones: those produced by adipose tissue (adipsin, leptin, visfatin, and PAI-1), those produced by the gut (GIP, GLP-1, and ghrelin), and those produced by immune cells (resistin, IL-6, IL-10, and IL-1RA). While univariable MR analysis estimates the total (i.e. indirect and direct) effect of each cytokine on the outcomes, multivariable MR estimates the direct effect of each cytokine on the outcomes.

Multivariable 2SLS analyses for HOMA-S and HOMA-B were also performed using the *AER* R package, which first regressed the cytokines and hormones per group (adipose, gut, immune) on the genetic variants in a multivariate multiple linear regression. In the second stage, the HOMA outcome was regressed linearly on the fitted values of each of the cytokines and hormones. We additionally performed causal mediation analysis using the *mediation* R package on any statistically significant cytokines or hormone to examine whether their effect on HOMA-S and HOMA-B is mediated through BMI [37].

We used the mr_mvivw function from the *MendelianRandomization* R package to perform multivariable MR for the T2D outcome via the IVW method using a fixed effects model. We performed similar sensitivity analyses as for the univariable MR analyses: the IVW method using a random effects model, the multivariable MR-Egger method, and the multivariable median-based method.

## Results

### Characteristics of the study population

Out of the total 5066 individuals, 2790 were non-T2D individuals and 2276 were affected by T2D (Table 1). As expected, those affected by T2D were older on average and had a higher mean BMI. Median levels of the circulating cytokines and hormones were higher in those with T2D compared with non-T2D individuals for all cytokines and hormones except resistin, which had similar levels between T2D and non-T2D individuals, and IL-10, which had lower levels in those with T2D. This is expected for IL-10 given its anti-inflammatory properties.

**Table 1:** Characteristics of the study population

	**Total *N***	**Non-T2D individuals (*N* = 2790)**	**Individuals with T2D (*N* = 2276)**
**Covariates**			
Age (years)	5066	45.6 ± 15.4	56.2 ± 11.1
Sex (female)	5066	1674 (60.0%)	1401 (61.6%)
BMI (kg/m^2^)	5066	26.4 ± 6.0	27.7 ± 5.5
Site	5066		
Ghana		769 (27.6%)	592 (26.0%)
Nigeria		1656 (59.4%)	1274 (56.0%)
Kenya		365 (13.1%)	410 (18.0%)
**Cytokines and hormones**			
Adipsin (ng/ml)	4854	1067.2 (837.2–1584.6)	1207.6 (887.0–1967.4)
Leptin (ng/ml)	4914	3.5 (0.67–10.8)	4.3 (1.5–10.4)
Visfatin (ng/ml)	4853	2.3 (1.4–4.0)	2.7 (1.6–4.4)
PAI-1 (ng/ml)	4914	31.2 (22.6–44.8)	33.6 (23.9–49.9)
GIP (pg/ml)	4956	183.3 (123.4–285.0)	252.9 (163.1–425.8)
GLP-1 (pg/ml)	4913	221.4 (177.0–282.6)	236.8 (183.0–317.7)
Ghrelin (pg/ml)	4923	248.6 (143.1–508.0)	311.2 (159.6–630.8)
Resistin (ng/ml)	4894	5.0 (3.3–7.7)	5.0 (3.3–8.1)
IL-6 (pg/ml)	1330	1.06 (0.73–1.67)	10.5 (0.69–1.70)
IL-10 (pg/ml)	1095	10.1 (8.3–12.7)	8.0 (7.0–11.0)
IL-1RA (pg/ml)	1416	301.1 (219.5–428.3)	351.4 (254.9–519.3)
**Diabetes-related phenotypes**			
HOMA-S	2552	136.6 (78.3–248.9)	*NA*
HOMA-B	2552	90.2 (60.9–130.9)	*NA*
Glucose (mmol/L)	4994	4.7 (4.1–5.1)	8.2 (5.9–12.4)

Continuous variables are in means ± SD for normally distributed variables. Non-normally distributed variables are expressed in medians and (25th–75th percentile). Categorical variables are in *n* (percentages). HOMA-S and HOMA-B were only calculated for non-T2D individuals

*BMI* body mass index, *T2D* type 2 diabetes

### Association analysis of cytokines and hormones with T2D and HOMA measures

**Fig. 2:**
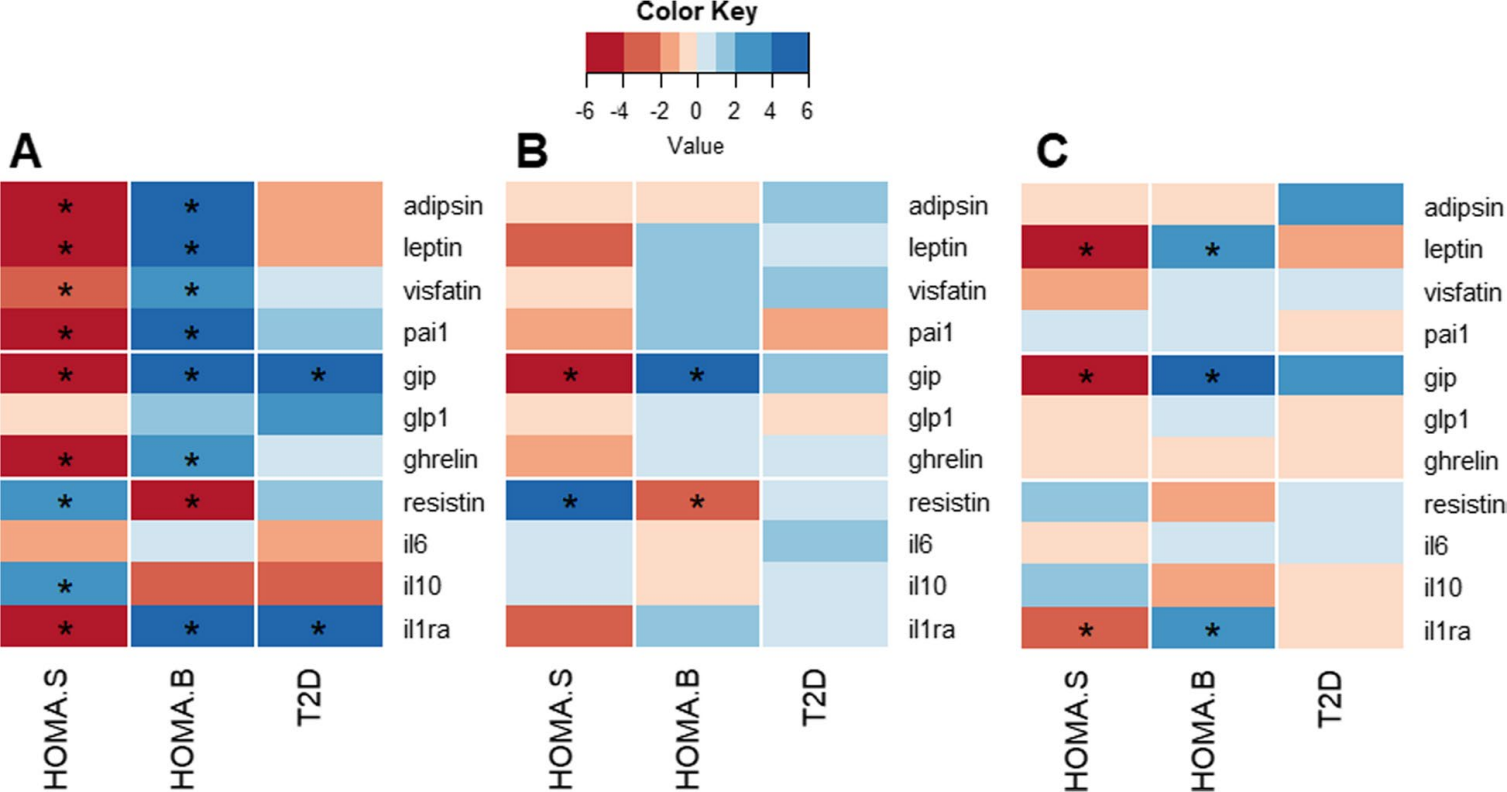
Heat map of **A** associations, **B** univariable MR causal associations, and **C** multivariable MR causal associations. The colour gradient reflects negative (red) and positive (blue) *Z* values for T2D and *T* values for the HOMA measures. HOMA analyses were performed in non-T2D individuals only. For T2D in panels **B** and **C**, the estimates from the inverse variance weighted (IVW) random effects model are shown. An asterisk indicates statistical significance at a Bonferroni-corrected *P*-value of < 0.0045 (= 0.05/11 cytokines and hormones)

Most cytokines and hormones were associated with the HOMA measures, the proxies for the hallmarks of T2D, insulin sensitivity (HOMA-S), and β-cell function (HOMA-B). All cytokines and hormones except for GLP-1 and IL-6 were associated with HOMA-S (Fig. 2A, Table 2). Resistin and IL-10 had a positive association with HOMA-S, while other cytokines and hormones had an inverse association with HOMA-S. GLP-1, IL-6, and IL-10 were not associated with HOMA-B at a Bonferroni-corrected *P*-value of 0.0045 (0.05/ 11 cytokines and hormones). The reverse of HOMA-S was seen for HOMA-B, with inverse associations for IL-10 and resistin and positive associations for all other cytokines and hormones.

**Table 2:** Associations of circulating cytokines and hormones with insulin sensitivity (HOMA-S), β-cell function (HOMA-B) and T2D

Cytokine/hormone	HOMA-S		HOMA-B		T2D	
	***β* (95% CI)**	***P*-value**	***β* (95% CI)**	***P*-value**	**OR (95% CI)**	***P*-value**
Adipsin	− 0.16 (− 0.20, − 0.11)	**1.07 × 10**^**−13**^	0.13 (0.09, 0.17)	**3.95 × 10**^**−10**^	0.95 (0.89, 1.02)	0.138
Leptin	− 0.30 (− 0.34, − 0.25)	**1.84 × 10**^**−35**^	0.27 (0.22, 0.31)	**7.94 × 10**^**−29**^	0.95 (0.87, 1.03)	0.245
Visfatin	− 0.06 (− 0.10, − 0.03)	**1.04 × 10**^**−3**^	0.07 (0.03, 0.11)	**5.28 × 10**^**−4**^	1.01 (0.95, 1.08)	0.610
PAI-1	− 0.13 (− 0.17, − 0.09)	**1.74 × 10**^**−10**^	0.12 (0.08, 0.16)	**1.39 × 10**^**−9**^	1.05 (0.99, 1.12)	0.089
GIP	− 0.16 (− 0.20, − 0.12)	**2.27 × 10**^**−16**^	0.13 (0.10, 0.17)	**6.61 × 10**^**−12**^	1.56 (1.46, 1.67)	**6.37 × 10**^**−38**^
GLP-1	− 0.02 (− 0.06, 0.02)	0.421	0.03 (− 0.01, 0.07)	0.140	1.07 (1.01, 1.14)	0.034
Ghrelin	− 0.08 (− 0.12, ^**−**^0.04)	**5.25 × 10**^**−5**^	0.07 (0.02, 0.10)	**2.11 × 10**^**−3**^	1.02 (0.96, 1.09)	0.471
Resistin	0.07 (0.03, 0.11)	**2.71 × 10**^**−4**^	− 0.08 (− 0.12, − 0.04)	**3.23 × 10**^**−5**^	1.03 (0.97, 1.10)	0.298
IL-6	− 0.04 (− 0.10, 0.021)	0.226	0.03 (− 0.04, 0.09)	0.430	0.91 (0.80, 1.02)	0.115
IL-10	0.11 (0.05, 0.17)	**4.26 × 10**^**−4**^	− 0.08 (− 0.15, − 0.02)	0.011	0.82 (0.70, 0.97)	0.019
IL-1RA	− 0.19 (− 0.26, − 0.13)	**1.06 × 10**^**−8**^	0.15 (0.09, 0.22)	**4.92 × 10**^**−6**^	1.34 (1.17, 1.52)	**1.03 × 10**^**−5**^

Models are adjusted for age, sex, BMI, and recruitment country. HOMA analyses were performed in non-T2D individuals only. Bold indicates significance at a Bonferroni-corrected *P*-value of 0.0045 (= 0.05/11 cytokines/hormones)

Two out of the eleven cytokines and hormones were associated with T2D, namely GIP and IL-1RA (Fig. 2A, Table 2). One unit increase in ORQ transformed GIP levels was associated with 1.56 times higher odds for T2D (*P*-value = 6.37 × 10^−38^) and one unit higher levels of ORQ transformed IL-1RA was associated with 1.34 higher odds (*P*-value = 1.03 × 10^−5^).

### Mendelian randomization analysis

A total of 35 SNPs were identified as genetic instruments for the eleven cytokines and hormones (Additional file 1; Table S1). All cytokines and hormones had multi-SNP instruments, except for IL-6 (Table 3). The three core assumptions of instrumental variables in MR analyses are the relevance (instrument is associated with exposure), exchangeability (instrument is not associated with confounders), and exclusion restriction (instrument only influences outcome via exposure) assumptions. We calculated the F-statistic and adjusted R squared per instrument to evaluate the relevance assumption (Table 3). The cumulative F-statistic of the instruments of each of the cytokines and hormones ranged from 13.6 (leptin) to 74.8 (adipsin) which are all above the threshold of an F-statistic of 10 or higher to be considered acceptable for MR analysis. While the assumptions of exchangeability and exclusion restriction cannot be empirically tested, we undertook several approaches to minimize the risk of pleiotropy violating these assumptions. We tested for evidence of potential pleiotropy by regressing all genetic instruments on age, sex, and BMI in our dataset of sub-Saharan Africans. Three out of the 35 genetic instruments (rs80117394 [adipsin], rs854781 [adipsin], and rs146197730 [leptin]) were found to be associated with age, sex, or BMI at a nominal *P*-value of < 0.01 and therefore potentially pleiotropic. While including these potentially pleiotropic variants could bias the MR estimates, excluding them could result in a significant loss of power. We therefore conducted a sensitivity analysis that included only those instruments not associated with any of the tested confounders at a nominal *P*-value of < 0.01, as described in the guidelines for performing MR by Burgess et al. [38]. Given that eight SNPs were associated with other cytokines and hormones in addition to the cytokine/hormone they were selected as instruments for (Additional file 1; Table S1), we were also able to perform multivariable MR analyses in addition to the univariable MR analyses. Using the selected instruments, power to detect an association in MR analyses was higher for the binary outcome T2D (ranging from 0.34 to 1.00) than for the HOMA measures which were analysed in non-T2D individuals only (power ranging from 0.23 to 0.99) (Table 3).

**Table 3:** Genetic instruments for all cytokines and hormones at a 5 × 10^−7^ *p*-value threshold

Cytokine/hormone	Number of participants	Number of genetic instruments	Cumulative F-statistic	Variance explained (R^2^)	Power HOMA-S^a^	Power HOMA-B^a^	Power T2D^a^
Adipsin	4854	3	74.8	0.090	0.89	0.88	0.99
Leptin	4914	2	13.6	0.011	0.23	0.23	0.34
Visfatin	4853	2	19.9	0.017	0.28	0.28	0.49
PAI-1	4914	3	20.9	0.026	0.39	0.38	0.66
GIP	4956	7	15.4	0.042	0.57	0.58	0.86
GLP-1	4913	3	15.3	0.018	0.28	0.27	0.51
Ghrelin	4923	2	30.5	0.025	0.38	0.38	0.65
Resistin	4894	7	62.4	0.159	0.99	0.99	1.00
IL-6	1330	1	24.3	0.032	0.46	0.45	0.75
IL-10	1095	2	24.0	0.060	0.74	0.74	0.95
IL-1RA	1416	3	20.5	0.069	0.80	0.79	0.97

^a^Power to detect a *β* of 0.2 or an OR of 1.5 at an *α* of 0.05

#### Univariable Mendelian randomization analyses

**Fig. 3:**
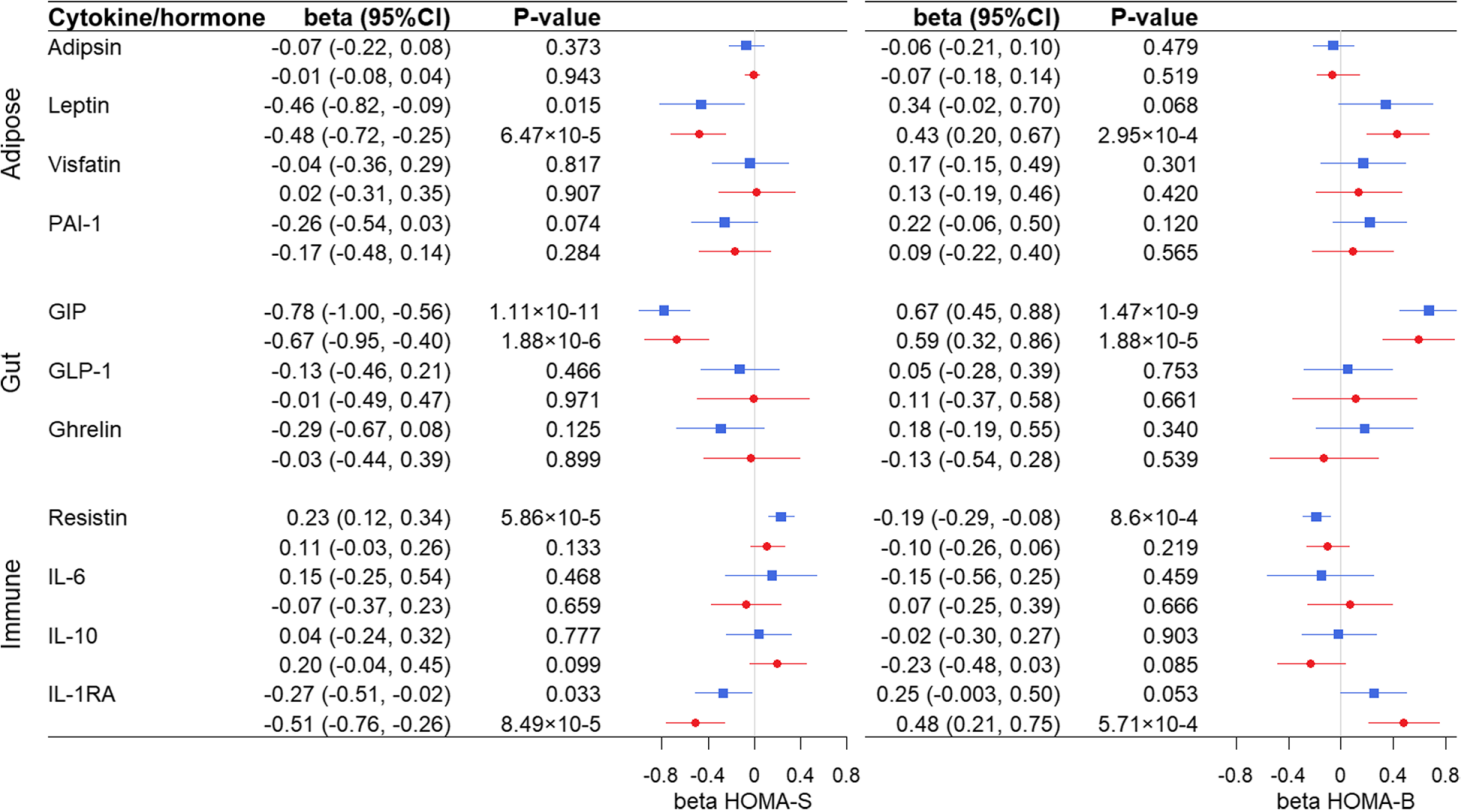
Forest plot of univariable (blue) and multivariable (red) MR causal associations of cytokines and hormones on HOMA-S (left) and HOMA-B (right). HOMA analyses were performed in non-T2D individuals only. A Bonferroni-corrected *P*-value of < 0.0045 (= 0.05/11 cytokines and hormones) was considered statistically significant

We found evidence for a causal effect of GIP and resistin on both HOMA-S and HOMA-B in the same direction as in the (traditional) association analysis using univariable 2SLS analyses (Figs. 2B and 3). Genetically predicted higher levels of GIP were associated with lower HOMA-S and with higher HOMA-B, while higher levels of resistin were associated with higher HOMA-S and lower HOMA-B (Fig. 3).

Our univariable MR results using the IVW fixed effects model with T2D as outcome suggested a causal effect of adipsin on T2D (Table 4). While the MR estimate using the median-based method was indicative of a causal effect as well, the association lost statistical significance when using the IVW random effects model and the MR-Egger method (Table 4, Fig. 2B). The *I*^2^ statistic (90.8%) and Cochran’s *Q* (21.8) for the fixed effects IVW adipsin model were suggestive of variation across genetic instruments (Table 4), which can also be seen in Additional file 1; Figure S1 with variant rs80117394 driving the association. We found two of the three genetic instruments for adipsin (rs80117394 and rs854781) associated with BMI at a nominal *P*-value of < 0.01. Hence, we performed an additional analysis excluding these variants as instruments. The IVW estimate for these conservative MR analyses was not statistically significant (OR = 0.61; 95%CI = 0.29, 1.25; *P*-value = 0.175). No evidence for a causal effect of any of the other cytokines or hormones on T2D was found (Table 4). The *I*^2^ statistics and Cochran’s *Q* were not indicative of heterogeneity for the other cytokines and hormones, except for IL-1RA (Table 4). For IL-1RA, we also observed a significant MR-Egger intercept, suggesting presence of pleiotropy.

**Table 4:** Univariable MR causal associations of cytokines and hormones on T2D

**Cytokine/hormone**	**IVW Fixed effects**		**IVW Random effects**		**Heterogeneity statistics**	
	**OR (95% CI)**	***P*-value**	**OR (95% CI)**	***P*-value**	**IVW Cochran’s Q (*P*-value)**	**IVW *I***^**2**^
Adipsin	**1.74 (1.44, 2.11)**	**8.46 × 10**^**−9**^	1.74 (0.93, 3.26)	0.081	21.81 (< 0.001)	90.8
Leptin	1.20 (0.73, 1.97)	0.470	1.20 (0.73, 1.97)	0.470	0.95 (0.330)	0.0
Visfatin	1.33 (0.87, 2.03)	0.181	1.33 (0.87, 2.03)	0.181	0.92 (0.338)	0.0
PAI-1	0.77 (0.53, 1.13)	0.185	0.77 (0.53, 1.13)	0.185	0.62 (0.735)	0.0
GIP	1.24 (0.96, 1.60)	0.104	1.24 (0.96, 1.60)	0.104	2.54 (0.864)	0.0
GLP-1	0.83 (0.54, 1.27)	0.391	0.83 (0.52, 1.34)	0.444	2.51 (0.285)	20.3
Ghrelin	1.09 (0.77, 1.56)	0.624	1.09 (0.65, 1.84)	0.738	2.15 (0.143)	53.5
Resistin	1.02 (0.89, 1.16)	0.821	1.02 (0.89, 1.16)	0.823	6.13 (0.409)	2.1
IL-6	1.19 (0.86, 1.65)	0.288	1.19 (0.86, 1.65)	0.288	*NA*	*NA*
IL-10	1.01 (0.82, 1.25)	0.903	1.01 (0.75, 1.37)	0.931	1.98 (0.159)	49.5
IL-1RA	1.00 (0.81, 1.25)	0.965	1.00 (0.63, 1.61)	0.984	9.18 (0.010)	78.2
	**Median**		**MR-Egger**		**Heterogeneity statistics**	
	**OR (95% CI)**	***P*-value**	**OR (95% CI)**	***P*-value**	**MR-Egger Intercept (*P*-value)**	**MR-Egger *I***^**2**^
Adipsin	**1.74 (1.40, 2.17)**	**6.81 × 10**^**−7**^	3.44 (0.81, 14.53)	0.093	− 0.225 (0.306)	97.0
Leptin	*NA*		*NA*			
Visfatin	*NA*		*NA*			
PAI-1	0.76 (0.48, 1.20)	0.237	0.41 (0.08, 2.06)	0.281	0.193 (0.433)	0.0
GIP	1.37 (0.98, 1.90)	0.062	1.41 (0.92, 2.14)	0.111	− 0.040 (0.446)	0.0
GLP-1	1.04 (0.59, 1.81)	0.894	0.30 (0.07, 1.29)	0.107	0.226 (0.155)	0.0
Ghrelin	*NA*		*NA*			
Resistin	1.03 (0.88, 1.20)	0.709	1.27 (0.93, 1.73)	0.131	− 0.108 (0.118)	95.2
IL-6	*NA*		*NA*			
IL-10	*NA*		*NA*			
IL-1RA	1.08 (0.79, 1.49)	0.621	2.52 (1.24, 5.12)	0.010	− 0.384 (0.007)	0.0

The MR-Egger and MR Median analyses could only be performed for cytokines with > 2 genetic instruments. Bold indicates significance at a Bonferroni-corrected *P*-value of < 0.0045 (= 0.05/11 cytokines/hormones)

#### Multivariable Mendelian randomization analyses

The causal effect of GIP on HOMA-S and HOMA-B that we observed in the univariable analyses was independent of other gut-produced hormones (namely, GLP-1 and ghrelin) and had a regression coefficient of − 0.67 for HOMA-S and 0.59 for HOMA-B (Figs. 2C and 3). In comparison with the univariable MR results, the multivariable estimate for resistin on both HOMA-S and HOMA-S was not statistically significant, suggesting that this association was not independent of other cytokines and hormones produced by immune cells. On the other hand, compared to the univariable MR findings, both leptin and IL-1RA gained statistical significance in the multivariable analyses for HOMA-S and HOMA-B (Figs. 2C and 3), suggesting that this cytokine and hormone may have a causal effect on insulin sensitivity and β-cell function independent of other cytokines and hormones. We performed a sensitivity analysis for adipose-produced cytokines and hormones by excluding the genetic instruments that were suggestive of potential pleiotropy (rs80117394, rs854781rs146197730). In this conservative approach, the multivariable MR estimates of leptin on HOMA-S (*β* =  − 0.50; 95%CI − 0.91, − 0.09; *P*-value = 0.016) and HOMA-B (*β* = 0.41; 95%CI − 0.01, 0.82; *P*-value = 0.045) lost Bonferroni-corrected statistical significance. Given the substantial role of adiposity in the development of glycaemic dysfunction and insulin resistance, we performed mediation analyses to explore whether the causal effect of GIP and IL-1RA may be partially mediated through BMI. We did not find evidence for BMI mediating the effect of GIP on HOMA-S (5.1%, 95%CI =  − 0.02, 0.12; *P*-value = 0.17), but BMI did mediate 30.8% of the effect of IL-1RA on HOMA-S (95%CI = 0.20, 0.45; *P*-value < 2 × 10^−16^). Similarly, BMI was not a mediator in the effect of GIP on HOMA-B (5.3%; 95%CI =  − 0.02, 0.13; *P*-value = 0.18), but it was a mediator in the effect of IL1-RA on HOMA-B (34%; 95%CI = 0.21, 0.53; *P*-value < 2 × 10^−16^).

The multivariable MR analyses for T2D showed that the statistically significant estimate of adipsin on T2D with the fixed effect IVW and median methods was independent of other cytokines and hormones produced by adipose tissue (Table 5). However, as with the univariable analyses, the IVW random effects and MR-Egger estimates were not significant, suggesting that the associations in the IVW fixed effects and median methods may be due to pleiotropy. In contrast to the univariable analyses, the leptin IVW fixed effects estimate was statistically significant in the multivariable analyses (Table 5). However, the lack of association in the sensitivity analyses suggests that this estimate is not robust. In addition, one of the two leptin genetic instruments (rs146197730) was associated with sex at a *P*-value of 0.006. The multivariable IVW estimate when excluding this potentially pleiotropic instrument was not statistically significant (*P*-value = 0.226). None of the other cytokines or hormones showed evidence of a causal effect on T2D independent of other cytokines and hormones.

**Table 5:** Multivariable MR causal associations of cytokines and hormones on T2D

Group	Cytokine/hormone	IVW Fixed effects		IVW Random effects		MR Egger		Median	
		**OR (95% CI)**	***P*-value**	**OR (95% CI)**	***P*-value**	**OR (95% CI)**	***P*-value**	**OR (95% CI)**	***P*-value**
Adipose	Adipsin	**1.83 (1.40, 2.39)**	**9.79 × 10**^**−6**^	1.83 (1.12, 3.00)	0.017	1.67 (0.56, 4.96)	0.357	**2.05 (1.27, 3.31)**	**0.003**
	Leptin	**0.56 (0.40, 0.78)**	**6.33 × 10**^**−4**^	0.56 (0.30, 1.04)	0.065	0.52 (0.18, 1.50)	0.226	0.39 (0.19, 0.81)	0.012
	Visfatin	1.01 (0.66, 1.53)	0.968	1.01 (0.46, 2.19)	0.983	0.96 (0.35, 2.61)	0.932	1.32 (0.53, 3.26)	0.546
	PAI-1	0.94 (0.62, 1.42)	0.760	0.94 (0.43, 2.03)	0.869	0.95 (0.40, 2.25)	0.909	0.89 (0.39, 2.04)	0.776
Gut	GIP	1.37 (1.04, 1.82)	0.026	1.37 (1.04, 1.82)	0.026	1.43 (0.97, 2.10)	0.070	1.41 (0.90, 2.21)	0.139
	GLP-1	0.88 (0.50, 1.54)	0.655	0.88 (0.50, 1.54)	0.655	0.89 (0.51, 1.56)	0.684	1.05 (0.49, 2.25)	0.908
	Ghrelin	0.97 (0.66, 1.42)	0.865	0.97 (0.66, 1.43)	0.865	0.97 (0.66, 1.42)	0.860	0.83 (0.47, 1.48)	0.527
Immune	Resistin	1.03 (0.89, 1.19)	0.724	1.03 (0.84, 1.26)	0.802	1.22 (1.00, 1.49)	0.047	1.03 (0.82, 1.30)	0.776
	IL-6	1.13 (0.84, 1.51)	0.423	1.13 (0.75, 1.70)	0.569	0.97 (0.70, 1.35)	0.868	1.33 (0.89, 1.99)	0.169
	IL-10	0.99 (0.80, 1.24)	0.953	0.99 (0.73, 1.36)	0.966	0.97 (0.77, 1.23)	0.824	1.11 (0.75, 1.64)	0.612
	IL-1RA	0.98 (0.78, 1.22)	0.826	0.98 (0.71, 1.33)	0.875	1.06 (0.83, 1.35)	0.642	0.59 (0.35, 0.99)	0.046

Bold indicates significance at a Bonferroni-corrected *P*-value of < 0.0045 (= 0.05/11 cytokines/hormones)

## Discussion

We examined the role of 11 circulating cytokines and hormones previously implicated in T2D in predominantly non-human studies. Circulating levels of nine out of the 11 cytokines and hormones were associated with insulin sensitivity and eight with β-cell function among non-T2D individuals. In addition, GIP and IL-1RA were associated with T2D. Furthermore, MR analyses provided evidence for an independent causal effect of circulating GIP levels on insulin sensitivity and β-cell function that was not mediated through BMI and an independent causal effect of IL-1RA on insulin sensitivity and β-cell function that was partially mediated through BMI. For all other cytokines and hormones, no robust evidence for a causal association with insulin sensitivity, β-cell function, or T2D was found.

The plausibility of a causal effect of fasting circulating GIP on reducing insulin sensitivity and promoting β-cell function is supported by evidence from other studies [39, 40]. GIP is an incretin secreted post-prandially by enteroendocrine K-cells found in the gastrointestinal tract, stomach, and pancreas [41]. GIP stimulates the release of insulin from pancreatic β-cells, which facilitates the storage and clearance of dietary triglycerides as well as adipose tissue expansion [41]. High-fat diets in mice were found to induce hypersecretion of GIP [42], and these increased GIP levels have been proposed to play an important role in the reduced insulin sensitivity that is observed in the presence of high-fat-diet consumption and elevated BMI [40, 41]. Despite GIP’s suggested role in insulin sensitivity in healthy individuals, GIP resistance has been observed in a T2D state when hyperglycaemia reduces GIP receptor expression in β-cells [43, 44]. In a prospective study of GIP and T2D incidence, fasting GIP levels were found elevated among normal glycaemic control individuals that developed T2D later [45]. Consistent with these prior observations, we found evidence for a causal effect on insulin sensitivity and β-cell function among non-T2D individuals, but no causal effect on T2D status. While GIP was not previously considered an attractive drug target, recent studies have started re-evaluating GIP’s therapeutic potential and have proposed GIP receptor signalling and dual GIP/GLP-1 receptor agonists as a novel means to reduce insulin resistance among T2D cases who have likely developed GIP resistance [46–48].

The role of GIP in insulin secretion is closely linked with GLP-1 but we found no evidence of a causal effect of circulating GLP-1 levels on insulin sensitivity, β-cell function, or T2D. In fact, neither circulating nor genetically predicted GLP-1 was significantly associated with any of these traits. This result may be a consequence of analysing fasting measures in this study as GLP-1 has a more potent action on postprandial insulin secretion in healthy individuals [44]. In addition, we note that a systematic review and meta-analyses of 22 trials found no difference in GLP-1 response between individuals with and without T2D [49]. Hence, our findings are consistent with the possibility that GLP-1 action is affected in T2D rather than GLP-1 circulating levels [49, 50]. Indeed, current GLP-1-based therapies for T2D are based on the activation of GLP-1 receptors through GLP-1 receptor agonists. GLP-1 has a short half-life and is degraded by the dipeptidyl peptidase 4 (DPP-4) enzyme. DPP-4 inhibitors are used in these GLP-1-based therapies to prevent degradation and inactivation of GLP-1 and prolong its action in improving glucose metabolism [47, 51].

The effect of IL-1RA on insulin sensitivity and β-cell function that we found in our study population of sub-Saharan Africans is in line with findings from some studies but in contrast with others. While MR analyses by Nowak et al. were in the same direction of effect as our findings, they did not find evidence for a causal association between IL-1RA and insulin sensitivity [52]. Neither did the Interleukin 1 Genetics Consortium [17] find associations of genetically elevated IL-1RA with insulin sensitivity or T2D. Both studies used data from predominantly European-ancestry populations. On the other hand, a SNP in the *P2RX7* gene was found to be associated with an increase in IL-1RA levels among T2D patients [53], which is consistent with our findings. Findings from randomized controlled trials that studied the effect of the drug Anakinra, which is a recombinant of the naturally occurring IL-1RA and binds to IL-1 receptors, also reported conflicting results with some finding IL-1RA to improve β-cell function [54], whereas others reporting no difference in insulin sensitivity after Anakinra treatment in obese non-T2D individuals [55]. In our analyses, we were only able to detect the causal association with insulin sensitivity and β-cell function in multivariable MR analyses that adjusted for confounding variables including other cytokines and hormones produced by immune cells, indicating the interplay between these cytokines and hormones and their potential to mask independent effects. Larger studies are needed to optimize IL-1RA genetic instruments for MR studies in diverse populations allowing for better understanding of whether IL-1RA plays a causal role in T2D and related traits.

If our findings of a causal effect of GIP and IL-1RA on insulin sensitivity and β-cell function are confirmed by other studies, there are potential implications for prevention of T2D development. Monitoring of circulating levels could serve as biomarkers for early detection and risk assessment as elevated levels of GIP and IL-1RA could signal increased risk for reduced insulin sensitivity and increased β-cell function. Increased β-cell function often precedes β-cell failure in T2D development [56]. In addition, it could drive further research in the development of novel therapies that target GIP and IL-1RA pathways [57]. There is a need for studies identifying modifiable lifestyle factors driving variation in GIP and IL-1RA levels so that these lifestyle factors can be targeted in intervention strategies among those at elevated risk for T2D.

Our null findings for other circulating cytokines and hormones are consistent with other MR studies that have investigated some of these cytokines and hormones in other populations. Wang et al. did not find evidence for a causal effect of leptin levels on T2D or HOMA measures using data from European-ancestry participants [16] and Song et al. found no evidence for causality of PAI-1 in T2D using SNPs in the *SERPINE1* gene as instrumental variables [58]. The causal effect of resistin levels in insulin sensitivity that our group and others reported previously [59, 60] was found to be dependent of other cytokines and hormones in our present multivariable MR analyses, which is consistent with other studies that evaluated *RETN* SNPs and did not find evidence for an effect of these SNPs on insulin sensitivity [61]. More broadly, Thériault et al. found only six out of 227 studied circulating proteins to be causally associated with blood pressure in a large MR study [62], suggesting most circulating proteins do not have causal roles in cardiometabolic pathologies.

Limitations of our analytic strategy are worth noting. Firstly, we employed a one-sample approach for the MR analyses with the HOMA measures as outcomes, which can lead to increased false positive findings in the presence of weak instruments. We attempted to mitigate this possibility by assessing F-statistics and excluding potentially problematic instruments. We note that no other genetic epidemiological cohort with data on the same circulating cytokines and hormones among sub-Saharan Africans exists. This situation presents a challenge considering instrumental variable assumptions are more likely to be violated when two samples represent different ethnic groups [38]. This also applies to the substantial differences in genetic make-up and environmental exposures between sub-Saharan African and African Americans. For adipsin, visfatin, and ghrelin, we are unaware of any other genetic epidemiological cohorts or GWAS summary statistics regardless of ancestry. Secondly, our relatively small sample size resulted in limited power to detect causal relationships for some cytokines and hormones as the power of an MR study increases with its sample size. Thirdly, measurement of the circulating cytokines and hormones in fasting blood samples could have obscured some associations. For example, GIP action differs between a fasting and postprandial state with stronger effects on glucagon and insulin secretion in a postprandial state [63]. Lastly, the generalizability of MR findings across multiple populations warrants careful investigation. There is a need for the inclusion in MR studies of diverse populations with diverse environmental exposures.

## Conclusions

In conclusion, this MR study using a sample of sub-Saharan Africans provides evidence for a causal effect of circulating GIP and IL-1RA levels on insulin resistance and β-cell function in non-T2D individuals and suggests that circulating levels of several other cytokines and hormones that have previously been reported in relation to T2D are not causal. While the effect of IL-1RA was partially mediated through BMI, the effect of GIP was not, suggesting that circulating GIP levels could be explored further as a potential biomarker for the development of insulin resistance. Given the few GWAS on circulating cytokines and hormones in general and among sub-Saharan African populations in particular, such studies are needed to expand and further validate these findings in sub-Saharan African and other populations.

### Supplementary Information

**Additional file 1. **Associations of individual genetic instruments with cytokines and hormones. Additional file 1 shows the effect sizes and *P*-values for the associations of all 35 genetic instruments with each of the 11 cytokines and hormones in Table S1. Figure S1 in this file shows the variant-specific estimates as well as the univariable inverse variance weighted (IVW), MR-Egger, and weighted mediation estimates for each of the 11 cytokines and hormones on type 2 diabetes as outcome.

## Data Availability

The dataset analysed during the current study is not publicly available due the informed consent obtained which does not grant permission for deposition in an open-access repository of research data. Qualified investigators can gain access to the data as part of a collaboration upon reasonable request consistent with the project’s IRB approval and signed informed consent by contacting the Principal Investigator of the AADM study, Dr. Charles Rotimi, at rotimic@mail.nih.gov.

## Abbreviations

2SLS: Two-stage least squares
AADM: Africa America Diabetes Mellitus Study
BMI: Body mass index
GIP: Glucose-dependent insulinotropic peptide
GLP-1: Glucagon-like peptide-1
GWAS: Genome-wide association study
HOMA-S: Homeostatic Model Assessment of Insulin Sensitivity
HOMA-B: Homeostatic Model Assessment of β-cell Function
IL-10: Interleukin 10
IL-1RA: Interleukin 1 receptor antagonist
IL-6: Interleukin 6
IVW: Inverse variance weighted
MR: Mendelian randomization
ORQ: Ordered quantile
PAI-1: Plasminogen activator inhibitor-1
SNP: Single-nucleotide polymorphism
T2D: Type 2 diabetes
