# Supplementary material for: Mendelian randomization analyses suggest a causal role for circulating GIP and IL-1RA levels in homeostatic model assessment-derived measures of β-cell function and insulin sensitivity in Africans without type 2 diabetes
Source: Genome Med. 2023 Dec 4;15:108. doi: 10.1186/s13073-023-01263-7 (PMC10694992; doi:10.1186/s13073-023-01263-7)
Supplement: Supplementary file 1 — Additional file 1. Associations of individual genetic instruments with cytokines and hormones. Additional file 1 shows the effect sizes and P-values for the associations of all 35 genetic instruments with each of the 11 cytokines and hormones in Table S1. Figure S1 in this file shows the variant-specific estimates as well as the univariable inverse variance weighted (IVW), MR-Egger, and weighted mediation estimates for each of the 11 cytokines and hormones on type 2 diabetes as outcome. [file 13073_2023_1263_MOESM1_ESM.pdf]

ADDITIONAL FILE 1: Associations of individual genetic instruments with cytokines and hormones

Table S1. Regression coefficients and *P*-values for genetic instruments and their association with cytokines and hormones in non-T2D individuals

**SNP (allele) Instrument's cytokine**	**Gene region**	**MAF**	**Adipsin**	**Leptin**	**Visfatin**	**PAI-1**	**GIP**	**GLP-1**	**Ghrelin**	**Resistin**	**IL-6**	**IL-10**	**IL-1RA**
rs537742646 (G)<br>Adipsin	*TPTE2P3*	0.025	**0.388**<br>**P=4.62E^{-5}**	0.119<br>P=0.256	0.061<br>P=0.536	0.101<br>P=0.278	-0.001<br>P=0.995	-0.123<br>P=0.18	-0.070<br>P=0.475	-0.011<br>P=0.907	0.015<br>P=0.938	-0.034<br>P=0.861	0.033<br>P=0.856
rs80117394 (A)<br>Adipsin	*KLC1*	0.219	**0.377**<br>**P=2.61E^{-41}**	-0.028<br>P=0.38	0.008<br>P=0.780	**0.195**<br>**P=2.26E^{-12}**	**-0.148**<br>**P=1.63E^{-7}**	**-0.224**<br>**P=1.26E^{-16}**	**0.345**<br>**P=3.77E^{-33}**	**-0.241**<br>**P=7.07E^{-18}**	-0.002<br>P=0.971	0.028<br>P=0.538	0.094<br>P=0.024
rs854781 (A)<br>Adipsin	*MYO15A*	0.320	**0.118**<br>**P=1.33E^{-4}**	**0.142**<br>**P=3.35E^{-4}**	0.05<br>P=0.123	0.018<br>P=0.547	0.032<br>P=0.301	-0.016<br>P=0.597	0.103<br>P=1.18E^{-3}	-0.026<br>P=0.405	0.017<br>P=0.763	-0.096<br>P=0.088	0.117<br>P=0.026
rs79770847 (G)<br>Leptin	IGR	0.074	-0.019<br>P=0.738	**-0.205**<br>**P=8.70E^{-4}**	-0.031<br>P=0.594	-0.017<br>P=0.752	-0.096<br>P=0.086	0.013<br>P=0.806	0.047<br>P=0.411	0.037<br>P=0.507	-0.124<br>P=0.232	-0.026<br>P=0.8	-0.147<br>P=0.134
rs146197730 (A)<br>Leptin	*TMEM182*	0.011	0.225<br>P=0.101	**0.602**<br>**P= 9.66E^{-5}**	-0.144<br>P=0.312	0.059<br>P=0.662	0.05<br>P=0.719	0.031<br>P=0.815	0.16<br>P=0.263	-0.034<br>P=0.805	0.136<br>P=0.568	-0.618<br>P=0.013	0.187<br>P=0.425
rs139705901 (A)<br>Visfatin	IGR	0.014	0.138<br>P=0.269	0.273<br>P=0.05	**-0.557**<br>**P=1.98E^{-5}**	-0.156<br>P=0.208	-0.207<br>P=0.103	-0.08<br>P=0.512	0.205<br>P=0.114	0.083<br>P=0.508	0.055<br>P=0.785	-0.206<br>P=0.302	0.145<br>P=0.437
rs78528426 (A)<br>Visfatin	IGR	0.095	0.103<br>P=0.037	0.022<br>P=0.686	**0.243**<br>**P=2.51E^{-6}**	0.059<br>P=0.225	**0.200**<br>**P=5.20E^{-5}**	0.068<br>P=0.153	0.019<br>P=0.708	0.048<br>P=0.332	0.257<br>P=5.05E^{-3}	-0.052<br>P=0.578	0.078<br>P=0.368
rs12288688 (A)<br>PAI-1	*TUB*	0.032	0.182<br>P=0.026	**0.318**<br>**P=4.57E^{-4}**	0.157<br>P=0.067	**0.415**<br>**P=2.34E^{-7}**	0.194<br>P=0.018	0.077<br>P=0.331	0.115<br>P=0.172	0.091<br>P=0.262	0.17<br>P=0.249	-0.104<br>P=0.482	**0.547**<br>**P=7.63E^{-5}**
rs141500779 (G)<br>PAI-1	IGR	0.052	0.189<br>P=0.004	0.141<br>P=0.052	-0.101<br>P=0.143	**-0.224**<br>**P=5.81E^{-4}**	-0.02<br>P=0.764	-0.077<br>P=0.231	0.056<br>P=0.408	-0.097<br>P=0.141	-0.166<br>P=0.124	-0.137<br>P=0.198	0.092<br>P=0.354
rs77829906 (G)<br>PAI-1	*EVA1C*	0.052	0.136<br>P=0.037	0.010<br>P=0.890	0.006<br>P=0.93	**0.289**<br>**P=5.68E^{-6}**	0.143<br>P=0.028	-0.108<br>P=0.087	-0.033<br>P=0.622	0.029<br>P=0.649	0.130<br>P=0.300	-0.103<br>P=0.418	0.056<br>P=0.625
rs11590440 (C)<br>GIP	IGR	0.013	0.206<br>P=0.114	0.230<br>P=0.107	0.001<br>P=0.993	0.206<br>P=0.102	**0.618**<br>**P=1.95E^{-6}**	0.136<br>P=0.274	0.078<br>P=0.554	0.104<br>P=0.417	0.582<br>P=0.195	-0.699<br>P=0.093	0.420<br>P=0.229
rs12045034 (C)<br>GIP	IGR	0.011	0.162<br>P=0.253	-0.032<br>P=0.833	0.122<br>P=0.386	-0.029<br>P=0.827	**0.568**<br>**P=3.3E^{-5}**	0.117<br>P=0.374	0.342<br>P=0.015	-0.363<br>P=0.008	0.619<br>P=0.082	0.091<br>P=0.8	0.297<br>P=0.369
rs182391695 (A)<br>GIP	*NUP155*	0.013	0.192<br>P=0.122	0.257<br>P=0.065	-0.086<br>P=0.503	0.154<br>P=0.21	**0.414**<br>**P=1.0E^{-3}**	0.292<br>P=0.015	**0.498**<br>**P=9.24E^{-5}**	-0.103<br>P=0.408	0.28<br>P=0.285	-0.020<br>P=0.94	0.061<br>P=0.805
rs770391162 (C)<br>GIP	IGR	0.101	0.086<br>P=0.072	0.077<br>P=0.144	0.056<br>P=0.257	0.078<br>P=0.098	**0.159**<br>**P=8.8E^{-4}**	-0.072<br>P=0.119	-0.028<br>P=0.572	-0.028<br>P=0.555	0.091<br>P=0.285	0.006<br>P=0.945	0.024<br>P=0.759
rs4909841 (T)<br>GIP	IGR	0.300	0.005<br>P=0.871	0.028<br>P=0.416	-0.014<br>P=0.675	0.041<br>P=0.187	**-0.105**<br>**P=8.0E^{-4}**	-0.042<br>P=0.161	-0.077<br>P=0.017	0.031<br>P=0.316	0.084<br>P=0.153	-0.033<br>P=0.568	-0.01<br>P=0.847
rs368966234 (C)<br>GIP	IGR	0.010	0.035<br>P=0.804	0.025<br>P=0.873	0.219<br>P=0.14	-0.033<br>P=0.816	**0.684**<br>**P=1.87E^{-6}**	0.304<br>P=0.029	0.294<br>P=0.045	-0.276<br>P=0.053	0.134<br>P=0.743	-0.259<br>P=0.448	-0.096<br>P=0.77
rs183288 (A)<br>GIP	IGR	0.081	0.028<br>P=0.593	0.114<br>P=0.047	0.088<br>P=0.103	0.051<br>P=0.323	**0.225**<br>**P=1.6E^{-5}**	0.053<br>P=0.296	0.036<br>P=0.499	0.009<br>P=0.865	-0.028<br>P=0.786	0.025<br>P=0.803	0.025<br>P=0.796

rs74130041 (G)<br>GLP-1	IGR	0.028	-0.062<br>P=0.49	-0.154<br>P=0.119	-0.083<br>P=0.372	-0.063<br>P=0.474	0.105<br>P=0.241	**-0.342**<br>**P=6.75E^{-5}**	**-0.413**<br>**P=6.14E^{-6}**	-0.164<br>P=0.066	-0.271<br>P=0.184	-0.131<br>P=0.536	-0.414<br>P=0.029
rs12245507 (C)<br>GLP-1	IGR	0.172	0.012<br>P=0.763	0.005<br>P=0.915	-0.048<br>P=0.236	-0.005<br>P=0.895	-0.065<br>P=0.096	**-0.168**<br>**P=6.52E^{-6}**	-0.064<br>P=0.110	-0.059<br>P=0.124	-0.019<br>P=0.781	-0.074<br>P=0.285	0.108<br>P=0.093
rs10403138 (G)<br>GLP-1	IGR	0.026	0.081<br>P=0.353	0.066<br>P=0.493	0.065<br>P=0.475	-0.058<br>P=0.501	0.047<br>P=0.596	**0.251**<br>**P=2.90E^{-3}**	**0.386**<br>**P=1.93E^{-5}**	0.049<br>P=0.572	-0.178<br>P=0.186	-0.258<br>P=0.065	0.036<br>P=0.784
rs188705037 (C)<br>Ghrelin	IGR	0.013	0.122<br>P=0.355	-0.034<br>P=0.812	-0.036<br>P=0.787	0.043<br>P=0.738	0.363<br>P=0.005	0.284<br>P=0.022	**0.623**<br>**P=2.56E^{-6}**	-0.084<br>P=0.513	0.717<br>P=0.044	0.268<br>P=0.409	0.209<br>P=0.504
rs9908703 (T)<br>Ghrelin	IGR	0.019	0.290<br>P=5.79E^{-3}	0.094<br>P=0.417	0.221<br>P=0.042	0.285<br>P=5.94E^{-3}	0.089<br>P=0.398	0.051<br>P=0.616	**0.653**<br>**P=9.17E^{-10}**	-0.190<br>P=0.068	0.025<br>P=0.886	0.07<br>P=0.689	-0.006<br>P=0.972
rs140404375 (T)<br>Resistin	IGR	0.114	-0.017<br>P=0.718	0.094<br>P=0.065	0.053<br>P=0.268	0.023<br>P=0.612	-0.036<br>P=0.43	0.013<br>P=0.765	0.052<br>P=0.268	**0.164**<br>**P=3.39E^{-4}**	0.058<br>P=0.477	-0.036<br>P=0.657	0.082<br>P=0.280
rs77027004 (A)<br>Resistin	*DPP6*	0.015	0.099<br>P=0.403	0.097<br>P=0.457	-0.179<br>P=0.143	-0.145<br>P=0.209	-0.147<br>P=0.22	-0.173<br>P=0.13	0.014<br>P=0.906	**-0.563**<br>**P=1.43E^{-6}**	-0.09<br>P=0.664	-0.291<br>P=0.160	-0.125<br>P=0.531
rs28453748 (T)<br>Resistin	IGR	0.045	0.158<br>P=0.024	0.124<br>P=0.107	0.054<br>P=0.458	0.158<br>P=0.021	**0.266**<br>**P=1.5E^{-4}**	-0.018<br>P=0.788	0.014<br>P=0.841	**-0.314**<br>**P=5.84E^{-6}**	0.157<br>P=0.331	0.058<br>P=0.723	0.259<br>P=0.086
rs116265962 (C)<br>Resistin	*STON2*	0.065	-0.17<br>P=0.004	-0.099<br>P=0.134	-0.12<br>P=0.054	-0.076<br>P=0.194	-0.017<br>P=0.780	-0.067<br>P=0.241	-0.121<br>P=0.049	**-0.251**<br>**P=2.29E^{-5}**	-0.212<br>P=0.046	0.023<br>P=0.83	-0.079<br>P=0.426
rs62113430 (T)<br>Resistin	*CAMSAP3*	0.117	0.025<br>P=0.573	0.088<br>P=0.072	-0.034<br>P=0.454	0.027<br>P=0.527	0.013<br>P=0.771	-0.020<br>P=0.643	0.032<br>P=0.482	**-0.355**<br>**P=3.44E^{-16}**	0.119<br>P=0.16	-0.048<br>P=0.566	0.08<br>P=0.307
rs3219175 (A)<br>Resistin	*RETN*	0.131	0.096<br>P=0.025	-0.018<br>P=0.705	0.054<br>P=0.229	0.016<br>P=0.707	-0.033<br>P=0.437	0.102<br>P=0.013	0.083<br>P=0.059	**0.686**<br>**P=2.76E^{-60}**	-0.167<br>P=0.024	0.065<br>P=0.375	-0.117<br>P=0.091
rs114184135 (A)<br>Resistin	*MCEMP1*	0.059	0.074<br>P=0.224	0.043<br>P=0.526	-0.047<br>P=0.458	-0.02<br>P=0.733	-0.040<br>P=0.518	-0.071<br>P=0.225	0.088<br>P=0.159	**-0.407**<br>**P=1.30E^{-11}**	0.129<br>P=0.246	-0.111<br>P=0.31	-0.015<br>P=0.887
rs6091971 (C)<br>IL-6	IGR	0.256	0.02<br>P=0.532	0.001<br>P=0.972	-0.063<br>P=0.063	-0.032<br>P=0.325	-0.092<br>P=4.78E^{-3}	0.024<br>P=0.451	0.023<br>P=0.485	0.003<br>P=0.939	**-0.284**<br>**P=1.01E^{-6}**	-0.018<br>P=0.761	-0.062<br>P=0.258
rs57830824 (A)<br>IL-10	*LOC124904258*	0.090	-0.038<br>P=0.459	-0.087<br>P=0.124	0.086<br>P=0.107	0.008<br>P=0.881	-0.053<br>P=0.297	-0.049<br>P=0.32	-0.089<br>P=0.089	-0.049<br>P=0.333	0.114<br>P=0.218	**0.424**<br>**P=7.43E^{-6}**	-0.017<br>P=0.843
rs113520822 (T)<br>IL-10	*NXPE2/ NXPE4*	0.020	-0.051<br>P=0.629	-0.016<br>P=0.89	0.066<br>P=0.544	0.059<br>P=0.57	0.074<br>P=0.486	0.036<br>P=0.719	0.018<br>P=0.868	0.071<br>P=0.496	0.334<br>P=0.163	**1.107**<br>**P=3.95E^{-7}**	0.192<br>P=0.358
rs76952475 (T)<br>IL-1RA	IGR	0.082	-0.027<br>P=0.604	-0.035<br>P=0.554	-0.048<br>P=0.379	0.021<br>P=0.687	-0.054<br>P=0.309	-0.052<br>P=0.306	-0.026<br>P=0.637	-0.003<br>P=0.952	-0.004<br>P=0.968	0.022<br>P=0.824	**0.431**<br>**P=2.94E^{-6}**
rs79964117 (G)<br>IL-1RA	IGR	0.180	-0.007<br>P=0.855	0.005<br>P=0.914	0.009<br>P=0.827	-0.006<br>P=0.876	0.031<br>P=0.417	-0.027<br>P=0.457	-0.012<br>P=0.756	0.037<br>P=0.332	0.078<br>P=0.267	0.034<br>P=0.628	**0.301**<br>**P=2.64E^{-6}**
rs543252070 (T)<br>IL-1RA	*KCNT1*	0.013	-0.129<br>P=0.321	-0.276<br>P=0.055	-0.397<br>P=0.004	-0.361<br>P=0.005	-0.270<br>P=0.04	-0.355<br>P=0.005	-0.080<br>P=0.545	-0.084<br>P=0.516	-0.235<br>P=0.301	-0.087<br>P=0.701	**-0.879**<br>**P=3.30E^{-5}**

IGR = Intergenic Region. Bold indicates significance at a Bonferroni-corrected P-value of 0.001 (=0.05/49 SNPs). The cytokine for which the instrument was selected is indicated in the first column.

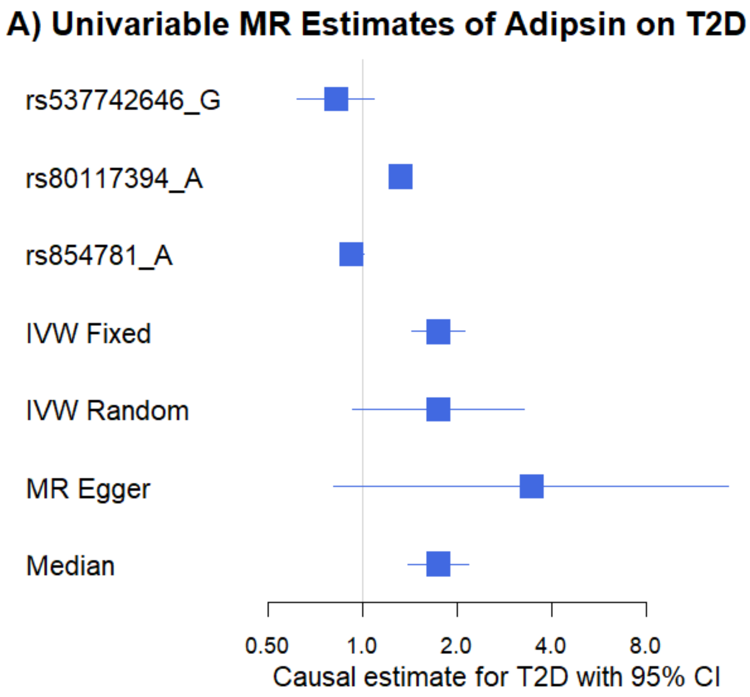
A) Univariable MR Estimates of Adipsin on T2D
rs537742646_G
rs80117394_A
rs854781_A
IVW Fixed
IVW Random
MR Egger
Median
0.50
1.0
2.0
4.0
8.0
Causal estimate for T2D with 95% CI

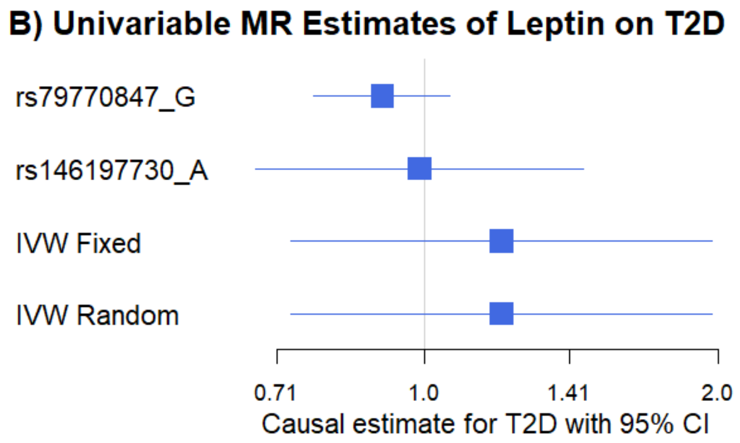
B) Univariable MR Estimates of Leptin on T2D
rs79770847_G
rs146197730_A
IVW Fixed
IVW Random
0.71
1.0
1.41
2.0
Causal estimate for T2D with 95% CI

C) Univariable MR Estimates of Visfatin on T2D

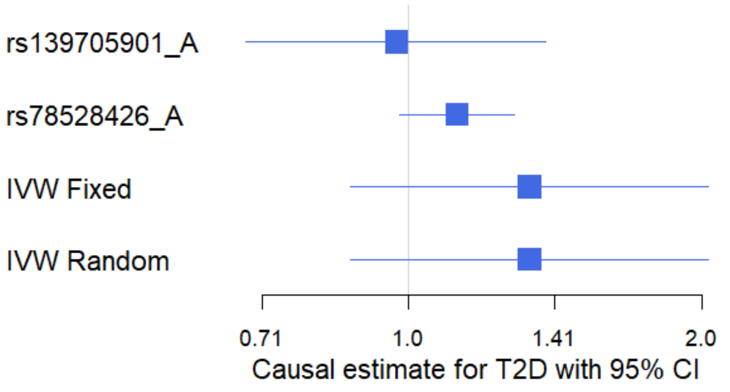

D) Univariable MR Estimates of PAI-1 on T2D

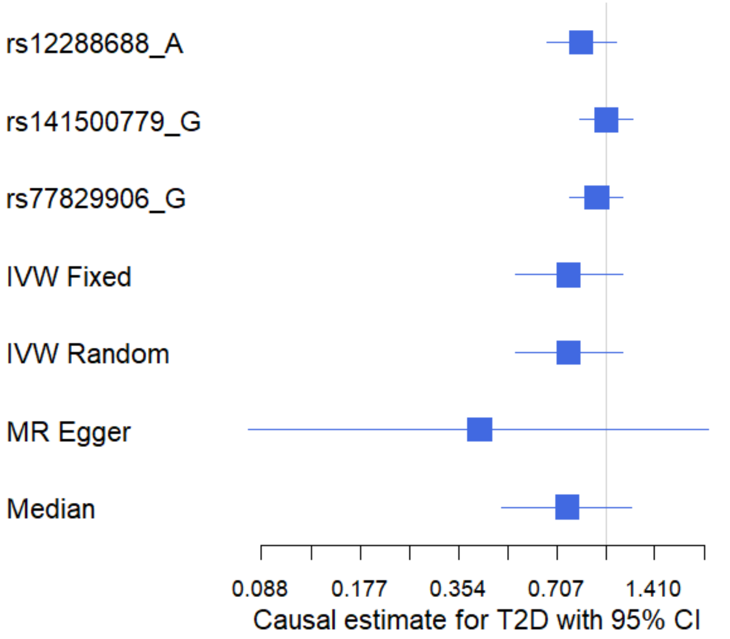

E) Univariable MR Estimates of GIP on T2D

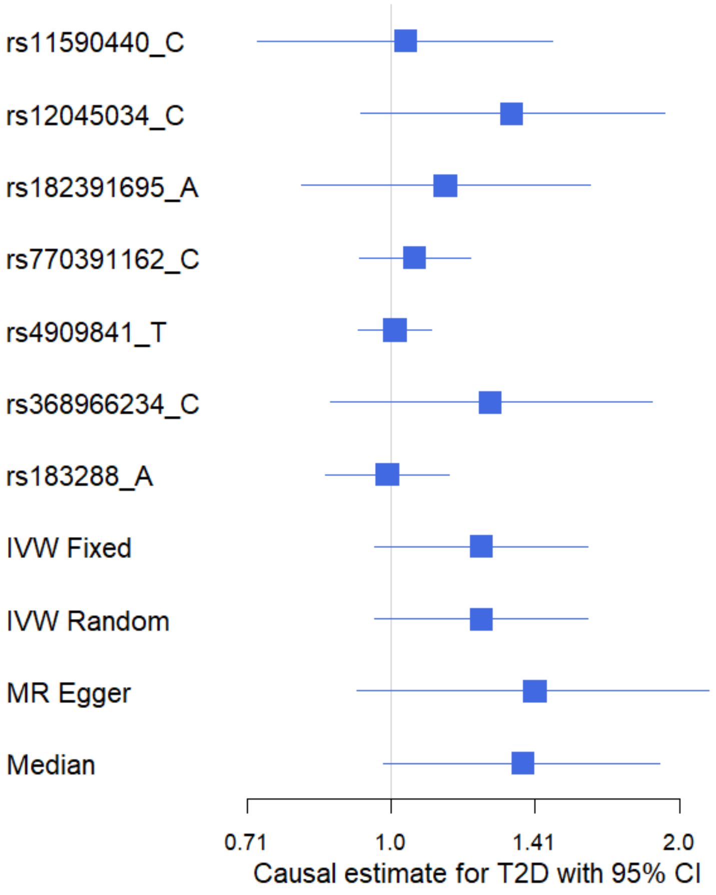

F) Univariable MR Estimates of GLP-1 on T2D

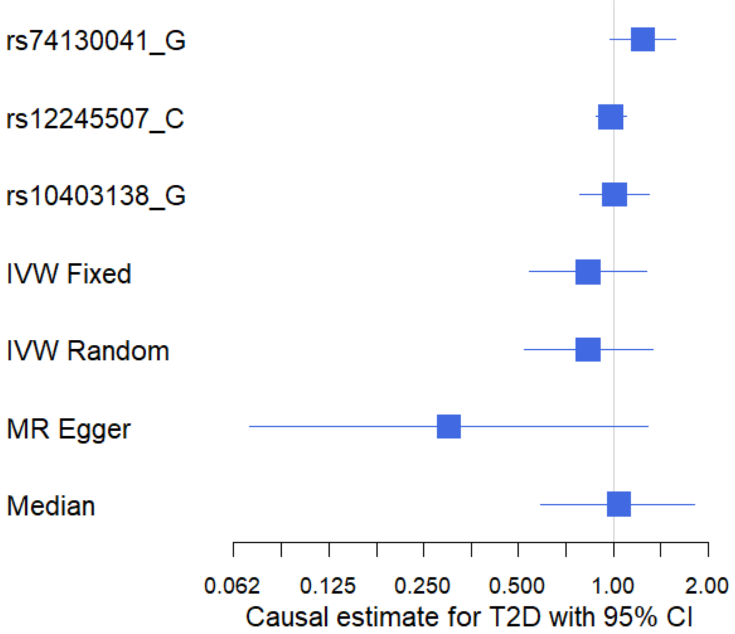

G) Univariable MR Estimates of Ghrelin on T2D

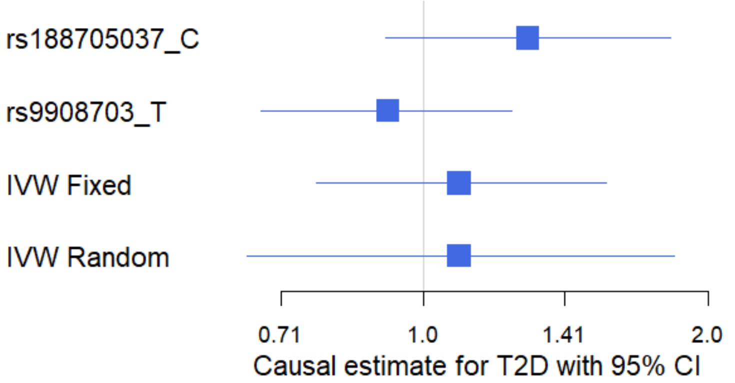

H) Univariable MR Estimates of Resistin on T2D

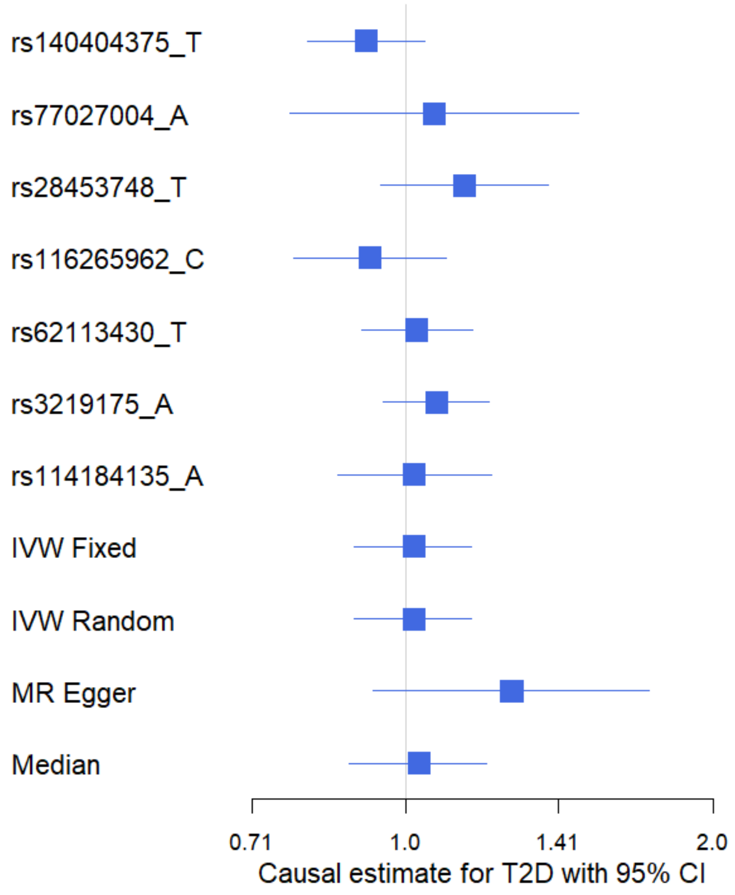

I) Univariable MR Estimates of IL-6 on T2D

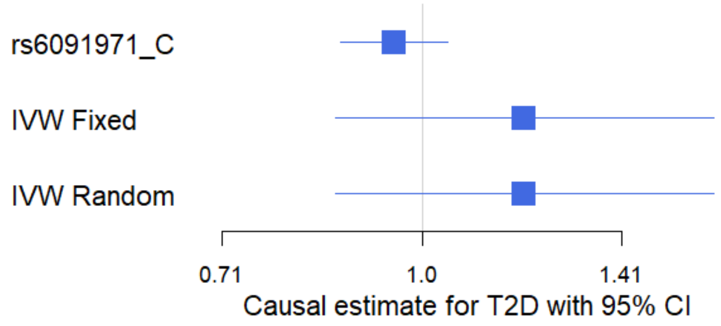

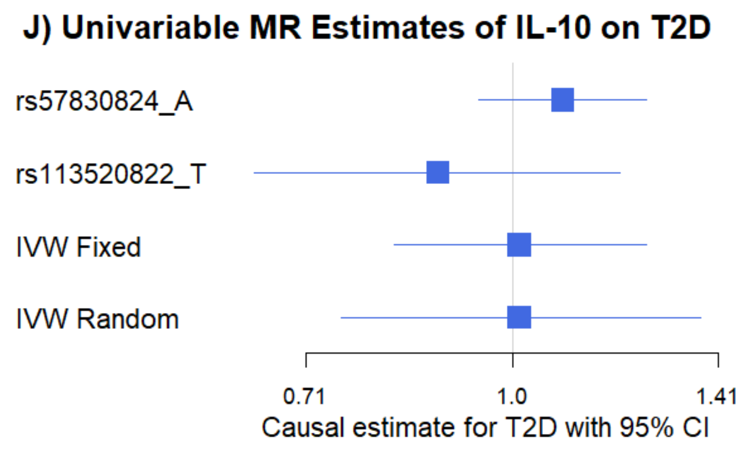

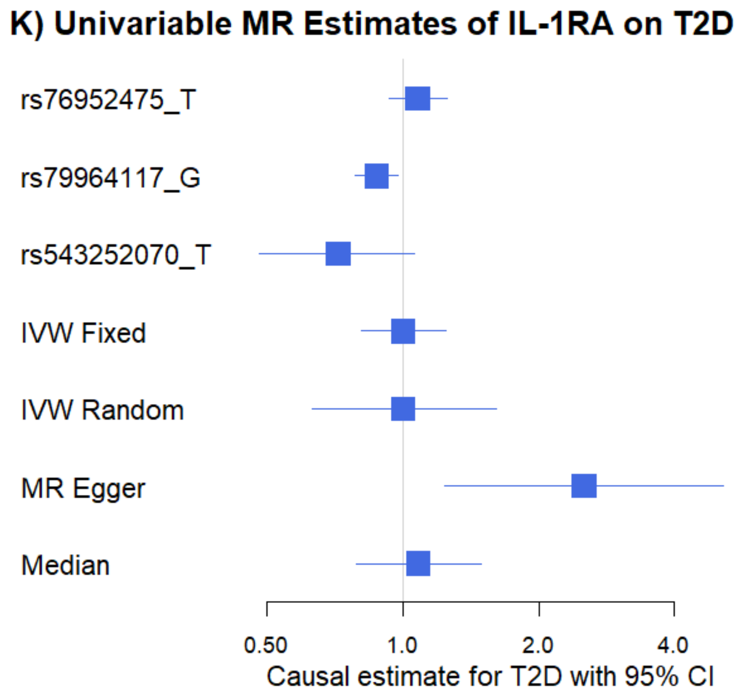

Figure S1. Forest plots showing variant-specific estimates and the Mendelian randomization (MR) inverse variance weighted (IVW) estimates for the fixed-effects and random-effects models. For cytokines and hormones with > 2 instruments, MR Egger and weighted median estimates are shown as well.
